# Declared funding and authorship by alcohol industry actors in the scientific literature: a bibliometric study

**DOI:** 10.1093/eurpub/ckaa172

**Published:** 2020-09-17

**Authors:** Su Golder, Jack Garry, Jim McCambridge

**Affiliations:** Department of Health Sciences, University of York, York, UK

## Abstract

**Background:**

Alcohol industry actors are known to be involved in scientific research. Despite concerns regarding bias, the extent of involvement and coverage of this research are unknown.

**Methods:**

We aimed to investigate the extent and type of scientific research 1918–2019 which was supported by the alcohol industry, including alcohol companies themselves and other organizations, such as trade associations. We identified bibliographic records from the Web of Science suite of databases which have named alcohol companies or organizations in the fields relating to author affiliations and support declarations. We then ascertained trends in publications over time, type of support, funding, outlets (such as journal titles), subject areas covered (such as health) and named companies (such as Carlsberg) and organizations (such as Drinkaware).

**Results:**

The analysis included 13 481 unique records, 11 014 (82%) were authored or funded by alcohol companies and 2488 (18%) were authored or funded by other organizations. The majority of the records (90%, 12 157/13 481) were journal publications. The most common subject areas covered by the publications were biology (5415/13 481, 40%), chemistry (3937/13 481, 29%) and health (3707/13 481, 27%). In line with general publishing trends, there has been an overall increase in research funded or supported by alcohol companies and organizations since records began. The main exception is the steady decline in company author affiliations, particularly in health-related topics since the mid-1990s.

**Conclusions:**

Alcohol companies and related organizations are extensively involved in or supporting scientific research according to data in Web of Science. This does not, however, necessarily reflect the totality of scientific research produced by alcohol companies and related organizations.

## Introduction

It is well known that drug trials undertaken by pharmaceutical companies selectively report outcomes and bias what is known and what is unknown. This has led to the rise of campaigns, such as Alltrials.net for more transparent reporting of the methods and results of trials, the development of trial registries and the release of industry data used for marketing approval.^1^ Robust evidence of bias extends to medical devices as well as for drugs.^2^ Nutrition research is afflicted by similar sources of bias, though the findings are less clear cut.^3–5^ Tobacco companies, on the other hand, have successfully conspired to subvert the scientific evidence-base for decades.^6^^,^^7^ Disclosure of conflicts of interests remains problematic in many areas.^8^^,^^9^ Such biases profoundly affect reviews and research agendas,^5^^,^^10–12^ as well as the findings of individual studies. Industry sponsorship of research thus poses major problems to the inferences that may be drawn safely from the scientific literature.

There has been little previous study of the extent of alcohol industry involvement in science.^13^ The ways in which alcohol industry actors use science to influence policy has been more extensively studied.^14^^,^^15^ One key scientific area that is highly policy relevant is the purported health benefits of alcohol, some of which are biologically implausible.^16^ Whilst the literature is evolving, there are possible cardioprotective effects of small amounts of alcohol that reverse as consumption increases.^17^^,^^18^ Industry actors have sponsored studies in this literature, and use any possible evidence of health benefits in efforts to influence policy.^14^ Reviews involving authors who have received industry funding appear more likely to identify cardioprotective effects.^19^ The only quantitative study to date identifies industry sponsorship effects in relation to findings for stroke and not heart conditions.^20^ Alcohol companies have recently funded the first clinical trial in this area, which was stopped because the biased nature of the study design was identified soon after the trial began.^21^

Whilst it may be difficult to uncover sources of funding or support for research, there has been increasing recognition of the importance of transparency about funding sources and conflicts of interest. This has led to the growing presence of funding acknowledgements in scientific publications, both published articles and conference proceedings.^22^^,^^23^ The study of acknowledgements as a source of data on research funding is now gaining momentum.^22–25^ However, there has been no previous study of the breadth of alcohol industry funding of research. Our aim is to narrow this research gap and to map the extent of scientific research which is conducted or supported by the alcohol industry.

Our research objectives are:

- to identify scientific research acknowledging direct funding or other support from alcohol industry actors including both alcohol companies themselves and related organizations, such as trade associations.
- to identify scientific research for which at least one of the authors is affiliated to an alcohol industry actor.
- to study the trends over time, output types, preferred journals and themes of this research, with a focus on health-related research.

## Methods

### Databases searched

In order to identify research supported or undertaken by alcohol companies or related organizations, we searched the Web of Science Core Collection (1900–present). Web of Science is a multidisciplinary suite of databases covering academic disciplines such as sciences, social science, arts and humanities. As of 2020, it contains more than 90 million articles from 1900 to the present day. The Web of Science Core Collection includes the following databases;

- Science Citation Index Expanded (SCI-EXPANDED) 1900–present,
- Sciences Citation Index (SSCI) 1956–present,
- Arts & Humanities Citation Index (A&HCI) 1975–present,
- Conference Proceedings Citation Index (CPCI-S) 1990–present,
- Social Science & Humanities (CPCI-SSH) 1990–present and
- Emerging Sources Citation Index (ESCI) 2015–present.

Web of Science was chosen because in 2008 Clarivate Analytics (then Thomson Reuters) initiated large-scale indexation of the funding acknowledgements collected in publications. These were made available for searching in Science Citations Index (SCI) in 2008, and in 2015 this practice was extended to Social Science Citation Index (SSCI) and Emerging Sources Citation Index. Studies have indicated that the acknowledgment information is erroneous in <1% of publications indexed on Web of Science despite being manually processed,^23^^,^^25^ though there is sometimes missing information such as country of funder or grant number.^22^

Web of Science also allows searching of organizations within the author field, which is preferable for more precise and accurate results than searching the complete ‘AD - Address’ field. This is because searches of the ‘AD - Address’ field search the complete author affiliation, including country, postal code, city, department or laboratory. For instance searching ‘Carlsberg’ as an organization as opposed to in the ‘AD - Address’ field will prevent the retrieval of articles authored by someone from ‘Dali Univ, *Carlsberg* Rd 32, Dali 671000, China’ or the ‘Univ Coll Capital, *Carlsberg* Campus, Humletorvet 3, Copenhagen, Denmark’.

### Search methods

Two searches were undertaken concurrently in Web of Science.

#### Search 1: author affiliation

For this search, we searched three fields. Firstly, ‘OG - Organization-Enhanced’- this field is indexed with the preferred names of companies or organizations which allows searching for the many variants of an institute’s name (such as Asahi Breweries, Asahi Brewery Co Ltd, Asahi Beer Pharmaceut Co Ltd, Asahi Breweries Co Ltd, Asahi Breweries Ltd). However, not all companies or organizations have been included in this list.

In addition, the fields ‘OO - Organization’ and ‘SG - Suborganization’ were searched as free-text fields with variant names of companies or organizations (Supplementary box S1).

#### Search 2: support acknowledgements

For this search, we searched two free-text fields, ‘FT - Funding Text’ and ‘FO - Funding Agency’. We used variant names of companies or related organizations (Supplementary box S2).

A ‘FT - Funding Text’ search retrieves information extracted from direct statements in anywhere in the full publication regarding *funding* or *support* such as in the ‘acknowledgments’ section or ‘conflicts/declarations of interest’ statement.

A ‘FO - Funding Agency’ search retrieves named organizations acknowledged in the full-text article for example, in the acknowledgement section or the source page.

No limits such as date, language or type of publication were applied to the searches.

### Selection of alcohol companies and related organizations

Alcohol companies and organizations were identified from existing data sources,^26–28^ from the controlled vocabulary in the ‘OG - Organization-Enhanced’ field and from preliminary searches. Since Web of Science databases do not include standard names or codes for funding organizations other than in the ‘OO - Organization-Enhanced’ field, variant names and acronyms were used where appropriate. Changes in names of both companies and related organizations were also accounted for as were translations into English of agencies named in other languages.

Some acronyms for industry-related organizations were removed from the search as sifting of the first 100 records indicated that they generated substantially more irrelevant results than relevant results. These acronyms were as follows:

SAIF (Self Regulating Alcohol Industry Forum), FRA (Fondation pour la Recherche en Alcoologie), FReD (Hong Kong Forum for Responsible Drinking), CISA (Centro de Informações sobre Saúde e Álcool), ARA (Industry Association for Responsible Alcohol Use), FAS (Fundacion Alcohol y Sociedad), FAAR (Foundation for Alcohol Related Research), AET (Alcohol Education Trust), DISCUS (Distilled Spirits Council of the United States), ICAP (International Center for Alcohol Policies), GODA (Foreningen Gode Alkoholdninger), VARD (Vietnam Association for Responsible Drinking), FEV (Spanish Wine Federation), FISAC (Fundacion De Investigaciones Sociales), ABFI (Alcohol Beverage Federation of Ireland), FEBE (Fundación Alcohol y Sociedad), SASPI (Society for Alcohol and Social Policy Initiative), IARD (International Alliance for Responsible Drinking), ISFAR (International Scientific Forum on Alcohol Research).

Whilst these include some key alcohol industry organizations, the full name in each case was searched for, and is most commonly used alone, or in combination with an abbreviation.

The industry organizations were either social aspects/public relations organizations (SAPROs) funded by alcohol companies^27^^,^^29^ or trade associations in which companies are members. Both types of organization may have the resources to fund research that could be published externally. These organizations may be registered as not-for-profits (charities) or as private companies.

### Data extraction

For both Searches 1 and 2, data were extracted on the named company/companies or related organization(s), the year of publication, the outlet type (journal, conference proceeding or book section), the journal title, the type of support received (financial, material or human resource) and the topic covered as assigned by Web of Science (such as ‘bioconservation’ or ‘oncology’).

The topic areas assigned by Web of Science were then coded to the most relevant over-arching subject areas by SG and JM. For example, records assigned by Web of Science to ‘Biochemistry & Molecular Biology’ and ‘Hematology’ were categorized under the subject areas ‘Biology’ and ‘Health’, respectively (Supplementary table S1).

In a few instances, no topic areas are assigned to the bibliographic record by Web of Science. Thus, for 251 records (2% of the total records, 251/13 591) a subject area (such as ‘Biology’) was assigned directly to the article by the author (SG) based on an assessment of the title and abstract. This meant that each record was then assigned to at least one subject area.

In Search 2, we identified different types of support declarations. We divided these into three categories based on whether the support was ‘monetary’, ‘materials’ or ‘human resource’. We defined ‘monetary’ support as direct funding including payment of expenses or salaries. For example, ‘This investigation was supported by a research grant (2007–2010) from the European Research Advisory Board’ or ‘Dr X salary was supported by a grant from Y’. The donation of ‘material’ category included examples such as ‘Ground wheat samples (together with Falling Number values for them) were provided by Campden BRI’ or ‘The authors wish to thank SABMiller plc for providing the commercial beer samples’. The last category—‘human resource’—was related to acknowledgments of assistance from individuals. Examples include, ‘We wish first and foremost to thank the many Carlsberg employees and executives who participated in our study’ or ‘Useful comments from Mr. Satoshi Ohara of Asahi Breweries are deeply appreciated’.

### Descriptive analysis

This analysis describes the main characteristics of the records found. We present data on the types of outlets, types of funding/support, time trends, named companies and organizations, subject coverage of research and present data on the records with an author affiliation and support declaration. We also provide separate data for the research with a health focus.

## Results

Searches were carried out on 18 June 2019. The author affiliation search (Search 1) resulted in 8520 records, of which 7318 were relevant. The support declarations search (Search 2) resulted in 7269 records of which 6613 were relevant. Some of the records were not relevant due to noise created by some search terms, for example, searching on ‘Guinness’ identified the ‘Guinness Eye Clinic’ and the ‘Guinness Book Records’, searching on ‘Carlsberg’ identified ‘Ny Carlsberg Glyptotek’, ‘Carlsberg Expeditions Syria’, and ‘Carlsberg Univ’, a search on ‘InBev’ identified ‘Chaire InBev’, a search on Anheuser-Busch identified ‘Anheuser-Busch Natural Resources Building’, a search on AWRI identified ‘Alberta Water Research Institute’, a search on ERAB identified authors with the initials ERAB, and a search on ‘Asahi’ identified ‘Asahi Soft Drinks Co Ltd’.

After exclusions, a total of 13 481 unique records were identified from Searches 1 and 2 and included in the analysis (see table 1). Only 450 (3%) records were identified in both Searches 1 and 2 that had both an author affiliation and a support declaration from an alcohol company/organization.

**Table 1:** Study reference type

	Journal article	Conference	Book section
Company author affiliation (*n* = 6053)	5075 (84%)	947 (16%)	31 (0.5%)
Company support declaration (*n* = 5138)	4938 (96%)	198 (4%)	2 (0.04%)
Any company support (11 014)	9843 (89%)	1138 (10%)	33 (0.3%)
Organization author affiliation (*n* = 1278)	1152 (90%)	122 (10%)	4 (0.3%)
Organization support declaration (*n* = 1493)	1463 (98%)	30 (2%)	0 (0%)
Any organization support (*n* = 2488)	2335 (94%)	149 (6%)	4 (0.2%)
**Total (*n* = 13** **481)**	**12** **157 (90%)**	**1287 (9%)**	**37 (0.3%)**

NB. Some references were identified by more than one search (e.g. company affiliation and company funding/support declaration)

The majority of all the records (81%, 11 014) were authored (6053) or supported (5138) by companies (such as Heineken). The other records were authored (1278) or supported (1493) by organizations (2488) (table 1). Only 21 records (0.2%) showed support from both a company and an organization.

### Most support for research was in the form of direct funding

Most of the support declarations in Search 2 pertained to direct financial support (94%, 6201/6613), with a few referring to non-financial support such as the provision of samples, materials or data (5%, 315/6613) and a smaller proportion acknowledging assistance in roles such as advisory board members from executives or other employees of alcohol companies or organizations (2%, 117/6613). A similar pattern emerged for both the companies and other organizations (Supplementary table S2).

### Most research was published in journal articles

Ninety percent (12 150/13 481) of the records referred to research published as journal articles, others were conference proceedings (9%) and a small proportion were book sections (0.3%) (table 1). References with support declarations (96% and 98%) were more likely to be in journal articles than those with a company or organization author affiliation (84% and 90%) (table 1). Author affiliations were more common in conference proceedings (16% and 10%) than support declarations (4% and 2%).

### Carlsberg dominates the individual companies

Overall, Carlsberg was by a considerable margin the most frequently appearing alcohol company in respect of both support declarations and author affiliations. This one company accounted for 87% (4480/5138) of all support declarations [with the majority from the ‘Carlsberg Foundation’ (84%, 4297/5138)] and for 40% (2396/6053) of author affiliations (with only 1.2%, 70/6053 affiliated to the ‘Carlsberg Foundation’).

The second most frequently appearing alcohol company was Kirin accounting for only (3%, 135/5138) of support declarations by companies, but nearly a third of author affiliations (32%, 1963/6053) among companies.

Overall, the most frequent organization in respect of funding declarations was the ‘Alcoholic Beverage Medical Research Foundation (ABMRF)’ (28%, 421/1493) followed by ‘The Australian Wine Research Institute (AWRI)’ (19%, 288/1493). By far, the most common author affiliation for organizations was ‘AWRI’ (70%, 896/1278).

When the analysis is restricted to those publications related to ‘Health’, it was found that with respect to publications with company support declarations Carlsberg still dominated (75%, 682/906) but Kirin dominated health research with company author affiliations (59%, 1041/17 554) followed by Carlsberg (16%, 275/1754).

With regards to support declarations in ‘Health’ related publications, the most common organizations were: ABMRF (43%, 397/916), ERAB (24%, 223/916) and IREB (11%, 98/916). The most common author affiliations for organizations of health-related publications were Campden Bri (24%, 50/205) and AWRI (41%, 84/2075).

### Increasing industry involvement in research over time

**Figure 1:**
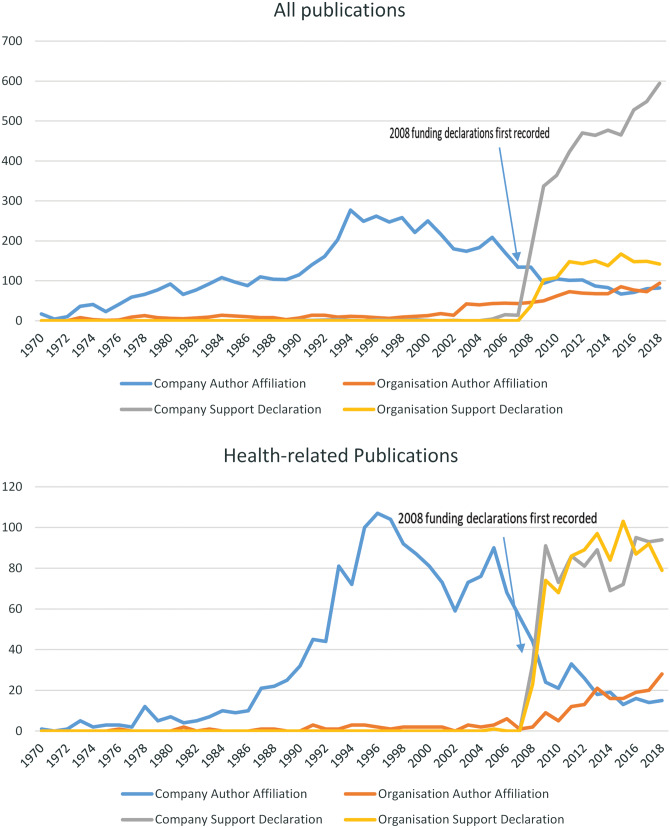
Time trends in publications from 1970

Since the initiation of funding data extraction in Web of Science in 2008, there has been a marked increase in company and organization declarations both for publications of all topics and for health-related publications (figure 1). However, support declarations by organizations appear much more prevalent with a steeper increase for health-related publications than for all publications (figure 1).

Author affiliations with organizations have also seen a slow steady increase from the 1970s to the present day both for publications of all topics and for health-related publications. However, author affiliations to companies appear to peak and then decline. The peak of research with company author affiliations is particularly pronounced for health-related publications with a first peak in the mid-1990s and a smaller second peak in the 2000s (figure 1).

### Most common topics areas and subjects

The topics assigned by Web of Science vary considerably in breadth making direct comparison of the numbers of records biased towards broader topic areas. However, the most common topics assigned by Web of Science were ‘Biochemistry & Molecular Biology’ (*n* = 2768), ‘Chemistry’ (*n* = 2465), ‘Food Science & Technology’ (*n* = 1901) and ‘Biotechnology & Applied Microbiology’ (*n* = 1287).

**Figure 2:**
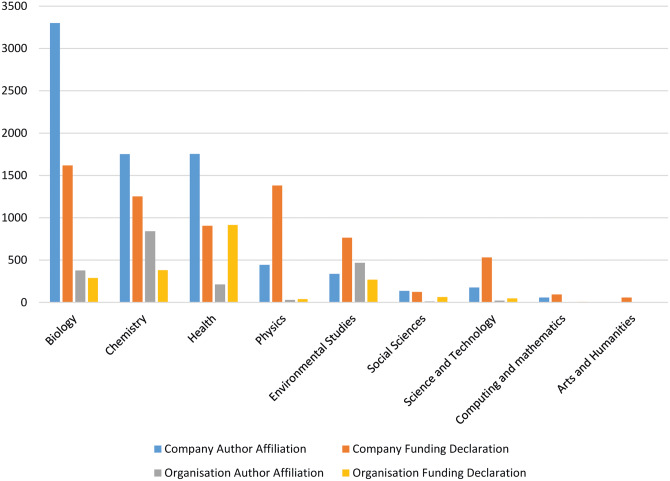
Subject areas covered by alcohol companies and related organizations

The subject areas in which alcohol industry actors (both companies and other organizations) have been most involved in are Biology (*n* = 5415), Chemistry (*n* = 3937) and Health (*n* = 3707) (figure 2). There was considerable variation in the subject areas covered depending on whether the support was from a company or an organization and depending on whether it was support via an author affiliation or a funding declaration. The most common subject areas in which companies were involved were Biology (*n* = 4836), Chemistry (*n* = 2928) and Health (*n* = 2630). The most common subject areas in which organizations were involved were Health (*n* = 1084), Chemistry (*n* = 1023) and Environmental Studies (*n* = 612).

The topics assigned by Web of Science were further explored for the health-related publications. The three most common health topics in publications with a company author affiliation were ‘Pharmacology & Pharmacy’ (22%, 380/1754), ‘Hematology’ (17%, 291/1754) and ‘Immunology’ (15%, 260/1754) and with a company support declaration were ‘Pharmacology & Pharmacy’ (25%, 223/906), ‘Physiology’ (13%, 116/906) and ‘Neurosciences & Neurology’ (12%, 107/906). The organizations, on the other hand, supported very different health topics to companies. Publications with an author affiliation by an organization were predominately on ‘Nutrition & Dietetics’ (50%, 100/201), ‘Genetics & Heredity’ (9%, 19/201) followed by ‘Substance Abuse’ (10%, 20/201). Those publications with support declarations by organizations were most commonly on the topic of ‘Substance Abuse’ (32%, 275/916), ‘Neurosciences & Neurology’ (21%, 194/916) and ‘Psychiatry’ (19%, 170/916).

### Most common journal titles were related to alcohol production and health

The journal articles were published in a wide range of titles, reflecting the topics covered. Again, there were variations in the most common journal titles according to whether the articles were associated with alcohol companies or related organizations, and also whether an author affiliation or support declaration was involved (table 2).

**Table 2:** Most common journal titles for health-related articles with alcohol company or organization author affiliation or support declaration

Journal title	Company author affiliation (%) (*n* = 1754)	Journal title	Company funding (%) (*n* = 906)	Journal title	Organization author xaffiliation (%) (*n* = 205)	Journal title	Organization funding (%) (*n* = 916)
*Blood*	133 (8)	Journal of Medicinal Chemistry	31 (3)	Food Chemistry	57 (28)	Alcoholism-Clinical and Experimental Research	78 (9)
*Journal of Antibiotics*	71 (4)	Journal of Medicinal Chemistry	27 (3)	British Medical Journal	9 (4)	Addictive Behaviors	40 (5)
*Journal of Immunology*	44 (3)	Food Chemistry	23 (3)	British Journal of Nutrition	7 (3)	Food Chemistry	46 (5)
*Experimental Hematology*	43 (2)	Blood	16 (2)	Proceedings of the Nutrition Society	7 (3)	Alcohol and Alcoholism	25 (3)
*Journal of Bone and Mineral Research*	36 (2)	Acs Chemical Neuroscience	13 (1)	Addiction	6 (2)	Drug and Alcohol Dependence	25 (3)
*Chemical Senses*	29 (2)	Chemmedchem	13 (1)	Bmc Genomics	5 (2)	Journal of Studies on Alcohol and Drugs	22 (2)

Publications with company author affiliations were commonly related to the alcohol industry or alcohol production [with the most common three journal titles being Carlsberg Research Communications (7%, 423/6053), Journal of the Institute of Brewing (3%, 175/6053) and Journal of the American Society of Brewing Chemists (3%, 156/6043)]. Publications with company support declarations, on the other hand, were more commonly published in general scientific journals (with the most common three journals being PLoS One (2%, 101/5138), Physical Review Letters (2%, 83/5138) and Physics Letters B (1%, 72/5138)).

Publications with organization author affiliations were commonly published in journal articles related to alcohol production [with the most common three journals being the Journal of Agricultural and Food Chemistry (13%, 167/1271), the Australian Journal of Grape and Wine Research (10%, 121/1271) and the American Journal of Enology and Viticulture (5%, 69/1271)]. However, those with support declarations by organizations were commonly related to alcohol production or addiction [with the most common three journals being the Journal of Agricultural and Food Chemistry (6%, 85/1493), Alcoholism-Clinical and Experimental Research (5%, 78/1493) and the Australian Journal of Grape and Wine Research (4%, 62/1493)].

An analysis was also undertaken of the most common journals in which health-related articles are published. Publications with company author affiliations were most commonly published in haematology and immunology journals whereas publications with support declarations from companies were published more widespread but most commonly in haematology and chemistry journals (table 2). Health-related publications with organization author affiliations or organization support declarations had a different focus. Health-related research with organization author affiliation tended to be published in nutrition or general health journals and health research with support declarations by organizations in journals related to addiction (table 2).

### Few instances where an article had both an author affiliation and a support declaration

There were only 450 instances where the record contained an author affiliation and a support declaration (i.e. was returned in both Search 1 and Search 2). This represents only 6% (450/7931) of the records published since 2008 (when Web of Science began recording funding data). Organizations were more likely to have both an author affiliation and a support declaration than companies (11%, 283/2488 vs. 2%, 177/11 014).

Those articles with both authorship affiliation and a support declaration were less likely to identify financial support (81%, 366/450 vs. 94%, 5835/6163) and more likely to have given support via materials (17%, 78/450 vs. 5%, 237/6163) than the main collection of records. There were similarly low proportions identifying assistance from individuals (3%, 17/450 vs. 2% 100/6163) (Supplementary table S2).

‘Environmental Studies’ and ‘Chemistry’ were more common subjects, and ‘Health’ less common in the duplicate records than the main collection of records (Supplementary table S3).

### Validation of the search methods

We tested the comprehensiveness of our search approach (Supplementary table S4). To do this an expert in the field, blind to our results, provided us with 10 papers in which the authors are known to have connections with the alcohol industry. We then sought whether each of the 10 references was identified by our search strategy. Of the 10 references, we retrieved half. Of those not retrieved, three were instances where the ‘conflict of interest’ in the full-text indicated links to industry but the ‘funding statements’ in Web of Science did not, such as reimbursement of the costs to present research or a fee received for proof reading a report. A further reference did not have any funding information in the full text paper. Lastly, one mentioned the ‘International Alliance for Responsible Drinking’ only as an acronym – ‘IARD’ and this was not searched for because of the large volume of non-relevant records retrieved when doing so.

## Discussion

This is the first bibliometric study of alcohol industry involvement in science. Findings are particularly illuminating on the scale, nature and breadth of alcohol industry funding of peer-reviewed research published in academic journals. Whilst there has been prior research attention to health topics,^21^ our findings suggest that the majority of alcohol industry support of research is not directly to do with health. Alcohol industry involvement in science, as captured by publications, is growing, and there are discernible trends in the nature of the activity.

Carlsberg dominates the research outputs. For the support declarations, this was mainly attributable to the Carlsberg Foundation, whilst for articles with author affiliation to Carlsberg this tended to be to the company itself. It appears from the volume of records that Carlsberg has a much stronger commitment to conducting and funding published research than other alcohol companies. It may also be the case that this particular company is more open than others. Further study of Carlsberg’s involvement in scientific research is needed. This is true also of the intriguing decline in author affiliations to companies since 1993.

The differences in the health topics covered by companies and organizations are of interest and may be of significant concern. Whilst companies tended to focus on more medical areas such as haematology and immunology, organizations tended to focus on addiction and behaviour. The relationship of organizations with industry may not be as apparent to the reader so this focus may cover industry influence which may focus on personal choice and thus personal blame. Further in-depth analysis of these articles may prove fruitful.

A rather profound limitation to the validity of study findings is that it is challenging to make strong inferences about how far the observed data reflects the extent of alcohol industry involvement in science, and on the nature of biases operating. It is difficult to ascertain the level of coverage of our searches and how much may have been missed. The validation study indicates that Web of Science has a strict definition of what constitutes funding/support with only direct funding and support considered applicable for the funding fields. We therefore need to address whether this is the tip of the iceberg, or whether the majority of relevant research has been identified. The lack of overlap between those records with an author affiliation and a support declaration, however, suggests that coverage may be far from complete. We are also aware that we excluded various acronyms, because they provided far more irrelevant records than relevant records and in the vast majority of instances the acronyms are not used in isolation, and among this group there are industry actors likely to be important.

We did not restrict our search by language; however, Web of Science only considers funding acknowledgements written in English.^23^ However, the greatest limitation is that we were unable to test how comprehensive our search was.

Beyond the bibliometric limitations, the most substantial grounds for concern about author disclosure of alcohol industry funding lies in the experience with the tobacco industry. Internal company documents made public following litigation demonstrate numerous methods of deception about funding information and other aspects of concealment, including lawyer vetting of papers to remove data linking the report to the company.^30–32^ The relevance of this information to the alcohol industry warrants careful consideration.^33^Cross-ownership between tobacco and alcohol companies appears particularly relevant to the extent to which alcohol companies adopt tobacco company strategies.^34–36^ It is significant that tobacco and alcohol companies have been working together recently to influence scientific norms and science policy in pursuit of their shared interests.^33^^,^^37^ Further, more detailed analysis of the dataset is also required to examine more closely trends in scientific research activities by different companies and organizations, and the content of the research. This dataset could be triangulated with other data sources in so doing. Further research could also examine the extent to which alcohol industry funding skews the research agenda, particularly in the health arena.

## Conclusions

Alcohol companies and related organizations are extensively involved in scientific research according to data in Web of Science. This does not, however, necessarily reflect the totality of scientific research produced by alcohol companies and related organizations. A number of questions for further research have been identified.

## Supplementary data

Supplementary data are available at *EURPUB* online.

## Supplementary Material

ckaa172_supplementary_data
