# Supplementary material for: Declared funding and authorship by alcohol industry actors in the scientific literature: a bibliometric study
Source: Eur J Public Health. 2020 Sep 17;30(6):1193–200. doi: 10.1093/eurpub/ckaa172 (PMC7733050; doi:10.1093/eurpub/ckaa172)
Supplement: ckaa172_supplementary_data [file ckaa172_supplementary_data.zip › ejph-2020-02-om-0159-File004.docx]

**Supplementary Information: Search Strategies**

**Box S1: Author Affiliation Search**

| #1 OG=((Anheuser-Busch InBev) OR AMBev OR (Asahi Beer Pharmaceut Co Ltd) Or (Asahi Breweries) Or (Asahi Breweries Co Ltd) Or (Asahi Breweries Ltd) Or (Asahi Brewery Co Ltd) Or (Asahi Grp Holdings) Or (Asahi Grp Holdings Ltd) OR (Asahi Group Holdings) OR (Asahi Group Holdings Ltd) OR (Australian Wine Research Institute) OR (Brown Forman) OR (Campden BRI) OR Carlsberg OR (Chivas Brothers) OR Diageo OR Drinkaware OR FIVS OR (Grand Metropolitan) OR Guinness OR Heineken OR Interbrew OR IREB OR (Kirin Holdings) OR (Kirin Brewery) OR MillerCoors OR (Molson Coors) OR (Pernod Ricard) OR SABMiller OR (Sapporo Holdings Limited) OR (South African Breweries)) (2307 hits)  #2 SG=((AB InBev) OR ABMRF OR ABRABE OR (Alcohol Awareness Foundation Ireland) OR (Alcohol Beverage Federation of Ireland) OR (Alcohol Beverages Australia) OR (Alcohol Education Trust) OR (Alcohol in Moderation) OR (Alcohol Research Group) OR (Alcoholic Beverage Medical Research Foundation) OR (Allied Domecq) OR AMBev OR (American Beverage Institute) OR (Amsterdam Group) OR ANEBE OR (Anheuser-Busch) OR (ANHEVSER BUSCH) OR APROCOR OR (Arnoldus Group) OR (Asahi Beer) OR (Asahi Breweries) OR (Asahi Brewery) OR (Asahi Group Holdings) OR ASDW OR (Asociación Gremial Chilena de Empresas de Bebidas Espirituosas Pro Consumo Responsible) OR (Associação Brasileira de Bebidas) OR (Associação Nacional de Bebidas Espirituosas) OR (Associação Nacional de Empresas de Bebidas Espirituosas) OR (Association of Small Direct Wine-Merchants) OR (Australian Wine Research Institute) OR AWRI OR Bacardi OR BBPA OR (Beam Suntory) OR (Beer and Health Europe) OR (Beer and Health Foundation) OR (Beer Canada) OR (Beer Institute) OR (Beer Selling Industry) OR (Beverages Company of the Americas) OR (Brewers Association) OR (Brewers of Europe) OR (Brewers Society) OR (Brewers' Society Medical Advisory Group) OR (British Beer and Pub Association) OR (Brown Forman) OR (Bulgarian Spirits Association) OR (Campden Brewing Research International) OR (Campden BRI) OR Carlsberg OR (Centro de Informações sobre Saúde e Álcool) OR (century council) OR (Cerveceros de México) OR (Cerveceros Latinoamericanos) OR (China Alcoholic Drinks Association) OR (Chivas Brothers) OR CIVB OR (Companhia de Bebidas das Américas) OR (Conseil Interprofessionnel du Vin de Bordeaux) OR (Council on Alcoholism and Drug Abuse) OR (Czech Spirits Association) OR (Deutsche Weinakademia) OR Diageo OR (Distilled Spirits Industry Council of Australia) OR Drinkaware OR Drinkwise OR DSICA OR (Distilled Spirits Council of the United States) OR Educ’alcool OR ERAB OR (European Confederation of Manufacturers of Spirits) OR (European Foundation for Alcohol Research) OR European Research Advisory Board OR (Federación Española de Bebidas Espirituosas) OR FIVIN OR FIVS OR (Fondation pour la Recherche en Alcoologie) OR (Foreningen Gode Alkoholdninger) OR (Foundacion de Investigaciones Sociales) OR (Foundation for Advancing Alcohol Responsibility) OR (Foundation for Alcohol Related Research) OR (Foundation for Alcohol Research) OR (Foundation for Responsible Alcohol Consumption) OR (French Federation of Spirits Drinks) OR (French Technical Institute of Wine) OR (French Wine and Vine Institute) OR (Fundacion Alcohol y Sociedad) OR (FUNDACION DE INVESTIGACIONES SOCIALES Alcohol-informate) OR (Fundación para la investigación del vino y nutrición) OR (Gannochy Trust) OR GAPG OR (Global Alcohol Producers Group) OR (God Alkoholkultur) OR (Grand Metropolitan) OR Guinness OR HAFRAC OR (Harm Reduction International) OR Heineken OR (Hong Kong Forum for Responsible Drinking) OR (Hungarian Association for Responsible alcohol consumption) OR (IFV F-30240 Le Grau Du Roi) OR (IFV, Le Grau du Roi) OR InBev OR (Industry Association for Responsible Alcohol Use) OR (Institut de Recherches Scientifiques sur les Boissons) OR (Institut Français de la Vigne et du Vin) OR Interbrew OR (Int Ctr Alcohol Policies) OR (International Alliance for Responsible Drinking) OR (International Center for Alcohol Policies) OR (International Scientific Forum on Alcohol Research) OR (International Spirits & Wines Association of India) OR (International Wine and Spirits Federation) OR IREB OR (Japanese Spirits and Liquor Makers Association) OR (Joseph E Seagram) OR (Joesph E Seagram) OR (Josepy E Seagram) OR (JE Seagram) OR JSLMA OR (Kirin Beverage Company) OR (Kirin Brewery) OR (Kirin Holdings) OR (Kirin Seagram) OR (Korea Alcohol and Liquor Industry Association) OR (Korea Alcohol Research Center) OR (Korea Alcohol Research Foundation) OR (Licensed Trade charity) OR (Miller Brewing Company) OR MillerCoors OR (Molson Coors) OR (National Association of Cider Makers) OR (Pernod Ricard) OR (Portman Group) OR (Renaud Society) OR (Research Foundation on Wine and Nutrition) OR (Robertson Trust) OR (Romanian Forum for Responsible Drinking) OR (SAB Miller) OR SABMiller OR (Sapporo breweries) OR (Sapporo beer) OR (Sapporo brew) OR (Sapparo brewing) OR (Sapparo wines) OR (Sapporom breweries) OR (Sapporo holdings) OR (Scotch Whisky Association) OR (“Scottish and Newcastle”) OR (Scottish Beer and Pub Association) OR (Seagram Lawrenceburg Distillery) OR (Sedex Global) OR (Self-Regulating Alcohol Industry Forum) OR (Society for Alcohol & Social Policy Initiative) OR (Society for Alcohol and Social Policy Initiative) OR (South African Breweries) OR (Spanish Federation of Spirits) OR (Spanish Wine Federation) OR (Spirits Canada) OR (Spirits New Zealand) OR SpiritsEurope OR (Stichting Verantwoorde Alcoholconsumptie) OR STIVA OR (Taiwan Beverage Alcohol Forum) OR TBAF OR TFRD OR (Thai Foundation for Responsible Drinking) OR (Union of Russian Brewers) OR (United Spirits) OR (US Brewers) OR UVDL OR (Vietnam Association for Responsible Drinking) OR (Vintners Charitable Foundation) OR (Wine and Spirit Trade Association) OR (Wine in moderation) OR (Wine Institute) OR (Wine World Trade group) OR (Women of Wine charities) OR (Worldwide Brewing Alliance) OR WSTA) (872 hits)  #3 OO=((AB InBev) OR ABMRF OR ABRABE OR (Alcohol Awareness Foundation Ireland) OR (Alcohol Beverage Federation of Ireland) OR (Alcohol Beverages Australia) OR (Alcohol Education Trust) OR (Alcohol in Moderation) OR (Alcohol Research Group) OR (Alcoholic Beverage Medical Research Foundation) OR (Allied Domecq) OR AMBev OR (American Beverage Institute) OR (Amsterdam Group) OR ANEBE OR (Anheuser-Busch) OR (ANHEVSER BUSCH) OR APROCOR OR (Arnoldus Group) OR (Asahi Beer) OR (Asahi Breweries) OR (Asahi Brewery) OR (Asahi Group Holdings) OR ASDW OR (Asociación Gremial Chilena de Empresas de Bebidas Espirituosas Pro Consumo Responsible) OR (Associação Brasileira de Bebidas) OR (Associação Nacional de Bebidas Espirituosas) OR (Associação Nacional de Empresas de Bebidas Espirituosas) OR (Association of Small Direct Wine-Merchants) OR (Australian Wine Research Institute) OR AWRI OR Bacardi OR BBPA OR (Beam Suntory) OR (Beer and Health Europe) OR (Beer and Health Foundation) OR (Beer Canada) OR (Beer Institute) OR (Beer Selling Industry) OR (Beverages Company of the Americas) OR (Brewers Association) OR (Brewers of Europe) OR (Brewers Society) OR (Brewers' Society Medical Advisory Group) OR (British Beer and Pub Association) OR (Brown Forman) OR (Bulgarian Spirits Association) OR (Campden Brewing Research International) OR (Campden BRI) OR Carlsberg OR (Centro de Informações sobre Saúde e Álcool) OR (century council) OR (Cerveceros de México) OR (Cerveceros Latinoamericanos) OR (China Alcoholic Drinks Association) OR (Chivas Brothers) OR CIVB OR (Companhia de Bebidas das Américas) OR (Conseil Interprofessionnel du Vin de Bordeaux) OR (Council on Alcoholism and Drug Abuse) OR (Czech Spirits Association) OR (Deutsche Weinakademia) OR Diageo OR (Distilled Spirits Industry Council of Australia) OR Drinkaware OR Drinkwise OR DSICA OR (Distilled Spirits Council of the United States) OR Educ’alcool OR ERAB OR (European Confederation of Manufacturers of Spirits) OR (European Foundation for Alcohol Research) OR European Research Advisory Board OR (Federación Española de Bebidas Espirituosas) OR FIVIN OR FIVS OR (Fondation pour la Recherche en Alcoologie) OR (Foreningen Gode Alkoholdninger) OR (Foundacion de Investigaciones Sociales) OR (Foundation for Advancing Alcohol Responsibility) OR (Foundation for Alcohol Related Research) OR (Foundation for Alcohol Research) OR (Foundation for Responsible Alcohol Consumption) OR (French Federation of Spirits Drinks) OR (French Technical Institute of Wine) OR (French Wine and Vine Institute) OR (Fundacion Alcohol y Sociedad) OR (FUNDACION DE INVESTIGACIONES SOCIALES Alcohol-informate) OR (Fundación para la investigación del vino y nutrición) OR (Gannochy Trust) OR GAPG OR (Global Alcohol Producers Group) OR (God Alkoholkultur) OR (Grand Metropolitan) OR Guinness OR HAFRAC OR (Harm Reduction International) OR Heineken OR (Hong Kong Forum for Responsible Drinking) OR (Hungarian Association for Responsible alcohol consumption) OR (IFV F-30240 Le Grau Du Roi) OR (IFV, Le Grau du Roi) OR InBev OR (Industry Association for Responsible Alcohol Use) OR (Institut de Recherches Scientifiques sur les Boissons) OR (Institut Français de la Vigne et du Vin) OR Interbrew OR (Int Ctr Alcohol Policies) OR (International Alliance for Responsible Drinking) OR (International Center for Alcohol Policies) OR (International Scientific Forum on Alcohol Research) OR (International Spirits & Wines Association of India) OR (International Wine and Spirits Federation) OR IREB OR (Japanese Spirits and Liquor Makers Association) OR (Joseph E Seagram) OR (Joesph E Seagram) OR (Josepy E Seagram) OR (JE Seagram) OR JSLMA OR (Kirin Beverage Company) OR (Kirin Brewery) OR (Kirin Holdings) OR (Kirin Seagram) OR (Korea Alcohol and Liquor Industry Association) OR (Korea Alcohol Research Center) OR (Korea Alcohol Research Foundation) OR (Licensed Trade charity) OR (Miller Brewing Company) OR MillerCoors OR (Molson Coors) OR (National Association of Cider Makers) OR (Pernod Ricard) OR (Portman Group) OR (Renaud Society) OR (Research Foundation on Wine and Nutrition) OR (Robertson Trust) OR (Romanian Forum for Responsible Drinking) OR (SAB Miller) OR SABMiller OR (Sapporo breweries) OR (Sapporo beer) OR (Sapporo brew) OR (Sapparo brewing) OR (Sapparo wines) OR (Sapporom breweries) OR (Sapporo holdings) OR (Scotch Whisky Association) OR (“Scottish and Newcastle”) OR (Scottish Beer and Pub Association) OR (Seagram Lawrenceburg Distillery) OR (Sedex Global) OR (Self-Regulating Alcohol Industry Forum) OR (Society for Alcohol & Social Policy Initiative) OR (Society for Alcohol and Social Policy Initiative) OR (South African Breweries) OR (Spanish Federation of Spirits) OR (Spanish Wine Federation) OR (Spirits Canada) OR (Spirits New Zealand) OR SpiritsEurope OR (Stichting Verantwoorde Alcoholconsumptie) OR STIVA OR (Taiwan Beverage Alcohol Forum) OR TBAF OR TFRD OR (Thai Foundation for Responsible Drinking) OR (Union of Russian Brewers) OR (United Spirits) OR (US Brewers) OR UVDL OR (Vietnam Association for Responsible Drinking) OR (Vintners Charitable Foundation) OR (Wine and Spirit Trade Association) OR (Wine in moderation) OR (Wine Institute) OR (Wine World Trade group) OR (Women of Wine charities) OR (Worldwide Brewing Alliance) OR WSTA) (7666 hits)  #4 #1 OR #2 OR #3 (8516 hits) |
| --- |

**Box S2: Funding fields search**

| #1 FO=((AB InBev) OR ABMRF OR ABRABE OR (Alcohol Awareness Foundation Ireland) OR (Alcohol Beverage Federation of Ireland) OR (Alcohol Beverages Australia) OR (Alcohol Education Trust) OR (Alcohol in Moderation) OR (Alcohol Research Group) OR (Alcoholic Beverage Medical Research Foundation) OR (Allied Domecq) OR AMBev OR (American Beverage Institute) OR (Amsterdam Group) OR ANEBE OR (Anheuser-Busch) OR (ANHEVSER BUSCH) OR APROCOR OR (Arnoldus Group) OR (Asahi Beer) OR (Asahi Breweries) OR (Asahi Brewery) OR (Asahi Group Holdings) OR ASDW OR (Asociación Gremial Chilena de Empresas de Bebidas Espirituosas Pro Consumo Responsible) OR (Associação Brasileira de Bebidas) OR (Associação Nacional de Bebidas Espirituosas) OR (Associação Nacional de Empresas de Bebidas Espirituosas) OR (Association of Small Direct Wine-Merchants) OR (Australian Wine Research Institute) OR AWRI OR Bacardi OR BBPA OR (Beam Suntory) OR (Beer and Health Europe) OR (Beer and Health Foundation) OR (Beer Canada) OR (Beer Institute) OR (Beer Selling Industry) OR (Beverages Company of the Americas) OR (Brewers Association) OR (Brewers of Europe) OR (Brewers Society) OR (Brewers' Society Medical Advisory Group) OR (British Beer and Pub Association) OR (Brown Forman) OR (Bulgarian Spirits Association) OR (Campden Brewing Research International) OR (Campden BRI) OR Carlsberg OR (Centro de Informações sobre Saúde e Álcool) OR (century council) OR (Cerveceros de México) OR (Cerveceros Latinoamericanos) OR (China Alcoholic Drinks Association) OR (Chivas Brothers) OR CIVB OR (Companhia de Bebidas das Américas) OR (Conseil Interprofessionnel du Vin de Bordeaux) OR (Council on Alcoholism and Drug Abuse) OR (Czech Spirits Association) OR (Deutsche Weinakademia) OR Diageo OR (Distilled Spirits Industry Council of Australia) OR Drinkaware OR Drinkwise OR DSICA OR (Distilled Spirits Council of the United States) OR Educ’alcool OR ERAB OR (European Confederation of Manufacturers of Spirits) OR (European Foundation for Alcohol Research) OR European Research Advisory Board OR (Federación Española de Bebidas Espirituosas) OR FIVIN OR FIVS OR (Fondation pour la Recherche en Alcoologie) OR (Foreningen Gode Alkoholdninger) OR (Foundacion de Investigaciones Sociales) OR (Foundation for Advancing Alcohol Responsibility) OR (Foundation for Alcohol Related Research) OR (Foundation for Alcohol Research) OR (Foundation for Responsible Alcohol Consumption) OR (French Federation of Spirits Drinks) OR (French Technical Institute of Wine) OR (French Wine and Vine Institute) OR (Fundacion Alcohol y Sociedad) OR (FUNDACION DE INVESTIGACIONES SOCIALES Alcohol-informate) OR (Fundación para la investigación del vino y nutrición) OR (Gannochy Trust) OR GAPG OR (Global Alcohol Producers Group) OR (God Alkoholkultur) OR (Grand Metropolitan) OR Guinness OR HAFRAC OR (Harm Reduction International) OR Heineken OR (Hong Kong Forum for Responsible Drinking) OR (Hungarian Association for Responsible alcohol consumption) OR (IFV F-30240 Le Grau Du Roi) OR (IFV, Le Grau du Roi) OR InBev OR (Industry Association for Responsible Alcohol Use) OR (Institut de Recherches Scientifiques sur les Boissons) OR (Institut Français de la Vigne et du Vin) OR Interbrew OR (Int Ctr Alcohol Policies) OR (International Alliance for Responsible Drinking) OR (International Center for Alcohol Policies) OR (International Scientific Forum on Alcohol Research) OR (International Spirits & Wines Association of India) OR (International Wine and Spirits Federation) OR IREB OR (Japanese Spirits and Liquor Makers Association) OR (Joseph E Seagram) OR (Joesph E Seagram) OR (Josepy E Seagram) OR (JE Seagram) OR JSLMA OR (Kirin Beverage Company) OR (Kirin Brewery) OR (Kirin Holdings) OR (Kirin Seagram) OR (Korea Alcohol and Liquor Industry Association) OR (Korea Alcohol Research Center) OR (Korea Alcohol Research Foundation) OR (Licensed Trade charity) OR (Miller Brewing Company) OR MillerCoors OR (Molson Coors) OR (National Association of Cider Makers) OR (Pernod Ricard) OR (Portman Group) OR (Renaud Society) OR (Research Foundation on Wine and Nutrition) OR (Robertson Trust) OR (Romanian Forum for Responsible Drinking) OR (SAB Miller) OR SABMiller OR (Sapporo breweries) OR (Sapporo beer) OR (Sapporo brew) OR (Sapparo brewing) OR (Sapparo wines) OR (Sapporom breweries) OR (Sapporo holdings) OR (Scotch Whisky Association) OR (“Scottish and Newcastle”) OR (Scottish Beer and Pub Association) OR (Seagram Lawrenceburg Distillery) OR (Sedex Global) OR (Self-Regulating Alcohol Industry Forum) OR (Society for Alcohol & Social Policy Initiative) OR (Society for Alcohol and Social Policy Initiative) OR (South African Breweries) OR (Spanish Federation of Spirits) OR (Spanish Wine Federation) OR (Spirits Canada) OR (Spirits New Zealand) OR SpiritsEurope OR (Stichting Verantwoorde Alcoholconsumptie) OR STIVA OR (Taiwan Beverage Alcohol Forum) OR TBAF OR TFRD OR (Thai Foundation for Responsible Drinking) OR (Union of Russian Brewers) OR (United Spirits) OR (US Brewers) OR UVDL OR (Vietnam Association for Responsible Drinking) OR (Vintners Charitable Foundation) OR (Wine and Spirit Trade Association) OR (Wine in moderation) OR (Wine Institute) OR (Wine World Trade group) OR (Women of Wine charities) OR (Worldwide Brewing Alliance) OR WSTA) (6,188 hits)  #2 FT=((AB InBev) OR ABMRF OR ABRABE OR (Alcohol Awareness Foundation Ireland) OR (Alcohol Beverage Federation of Ireland) OR (Alcohol Beverages Australia) OR (Alcohol Education Trust) OR (Alcohol in Moderation) OR (Alcohol Research Group) OR (Alcoholic Beverage Medical Research Foundation) OR (Allied Domecq) OR AMBev OR (American Beverage Institute) OR (Amsterdam Group) OR ANEBE OR (Anheuser-Busch) OR (ANHEVSER BUSCH) OR APROCOR OR (Arnoldus Group) OR (Asahi Beer) OR (Asahi Breweries) OR (Asahi Brewery) OR (Asahi Group Holdings) OR ASDW OR (Asociación Gremial Chilena de Empresas de Bebidas Espirituosas Pro Consumo Responsible) OR (Associação Brasileira de Bebidas) OR (Associação Nacional de Bebidas Espirituosas) OR (Associação Nacional de Empresas de Bebidas Espirituosas) OR (Association of Small Direct Wine-Merchants) OR (Australian Wine Research Institute) OR AWRI OR Bacardi OR BBPA OR (Beam Suntory) OR (Beer and Health Europe) OR (Beer and Health Foundation) OR (Beer Canada) OR (Beer Institute) OR (Beer Selling Industry) OR (Beverages Company of the Americas) OR (Brewers Association) OR (Brewers of Europe) OR (Brewers Society) OR (Brewers' Society Medical Advisory Group) OR (British Beer and Pub Association) OR (Brown Forman) OR (Bulgarian Spirits Association) OR (Campden Brewing Research International) OR (Campden BRI) OR Carlsberg OR (Centro de Informações sobre Saúde e Álcool) OR (century council) OR (Cerveceros de México) OR (Cerveceros Latinoamericanos) OR (China Alcoholic Drinks Association) OR (Chivas Brothers) OR CIVB OR (Companhia de Bebidas das Américas) OR (Conseil Interprofessionnel du Vin de Bordeaux) OR (Council on Alcoholism and Drug Abuse) OR (Czech Spirits Association) OR (Deutsche Weinakademia) OR Diageo OR (Distilled Spirits Industry Council of Australia) OR Drinkaware OR Drinkwise OR DSICA OR (Distilled Spirits Council of the United States) OR Educ’alcool OR ERAB OR (European Confederation of Manufacturers of Spirits) OR (European Foundation for Alcohol Research) OR European Research Advisory Board OR (Federación Española de Bebidas Espirituosas) OR FIVIN OR FIVS OR (Fondation pour la Recherche en Alcoologie) OR (Foreningen Gode Alkoholdninger) OR (Foundacion de Investigaciones Sociales) OR (Foundation for Advancing Alcohol Responsibility) OR (Foundation for Alcohol Related Research) OR (Foundation for Alcohol Research) OR (Foundation for Responsible Alcohol Consumption) OR (French Federation of Spirits Drinks) OR (French Technical Institute of Wine) OR (French Wine and Vine Institute) OR (Fundacion Alcohol y Sociedad) OR (FUNDACION DE INVESTIGACIONES SOCIALES Alcohol-informate) OR (Fundación para la investigación del vino y nutrición) OR (Gannochy Trust) OR GAPG OR (Global Alcohol Producers Group) OR (God Alkoholkultur) OR (Grand Metropolitan) OR Guinness OR HAFRAC OR (Harm Reduction International) OR Heineken OR (Hong Kong Forum for Responsible Drinking) OR (Hungarian Association for Responsible alcohol consumption) OR (IFV F-30240 Le Grau Du Roi) OR (IFV, Le Grau du Roi) OR InBev OR (Industry Association for Responsible Alcohol Use) OR (Institut de Recherches Scientifiques sur les Boissons) OR (Institut Français de la Vigne et du Vin) OR Interbrew OR (Int Ctr Alcohol Policies) OR (International Alliance for Responsible Drinking) OR (International Center for Alcohol Policies) OR (International Scientific Forum on Alcohol Research) OR (International Spirits & Wines Association of India) OR (International Wine and Spirits Federation) OR IREB OR (Japanese Spirits and Liquor Makers Association) OR (Joseph E Seagram) OR (Joesph E Seagram) OR (Josepy E Seagram) OR (JE Seagram) OR JSLMA OR (Kirin Beverage Company) OR (Kirin Brewery) OR (Kirin Holdings) OR (Kirin Seagram) OR (Korea Alcohol and Liquor Industry Association) OR (Korea Alcohol Research Center) OR (Korea Alcohol Research Foundation) OR (Licensed Trade charity) OR (Miller Brewing Company) OR MillerCoors OR (Molson Coors) OR (National Association of Cider Makers) OR (Pernod Ricard) OR (Portman Group) OR (Renaud Society) OR (Research Foundation on Wine and Nutrition) OR (Robertson Trust) OR (Romanian Forum for Responsible Drinking) OR (SAB Miller) OR SABMiller OR (Sapporo breweries) OR (Sapporo beer) OR (Sapporo brew) OR (Sapparo brewing) OR (Sapparo wines) OR (Sapporom breweries) OR (Sapporo holdings) OR (Scotch Whisky Association) OR (“Scottish and Newcastle”) OR (Scottish Beer and Pub Association) OR (Seagram Lawrenceburg Distillery) OR (Sedex Global) OR (Self-Regulating Alcohol Industry Forum) OR (Society for Alcohol & Social Policy Initiative) OR (Society for Alcohol and Social Policy Initiative) OR (South African Breweries) OR (Spanish Federation of Spirits) OR (Spanish Wine Federation) OR (Spirits Canada) OR (Spirits New Zealand) OR SpiritsEurope OR (Stichting Verantwoorde Alcoholconsumptie) OR STIVA OR (Taiwan Beverage Alcohol Forum) OR TBAF OR TFRD OR (Thai Foundation for Responsible Drinking) OR (Union of Russian Brewers) OR (United Spirits) OR (US Brewers) OR UVDL OR (Vietnam Association for Responsible Drinking) OR (Vintners Charitable Foundation) OR (Wine and Spirit Trade Association) OR (Wine in moderation) OR (Wine Institute) OR (Wine World Trade group) OR (Women of Wine charities) OR (Worldwide Brewing Alliance) OR WSTA) (7229 hits)  #3 #1 OR #2 (7,235) |
| --- |
