# Supplementary material for: Declared funding and authorship by alcohol industry actors in the scientific literature: a bibliometric study
Source: Eur J Public Health. 2020 Sep 17;30(6):1193–200. doi: 10.1093/eurpub/ckaa172 (PMC7733050; doi:10.1093/eurpub/ckaa172)
Supplement: ckaa172_supplementary_data [file ckaa172_supplementary_data.zip › ejph-2020-02-om-0159-File005.docx]

**Supplementary Table S1: Topic areas assigned by Web of Science and corresponding subject areas**

| **Topic Area assigned by Web of Science** | **Subject Area assigned by SG** |
| --- | --- |
| Acoustics, Astronomy & Astrophysics, Biophysics, Construction & Building Technology, Energy & Fuels, Imaging Science & Photographic Technology, Instruments & Instrumentation, Engineering, Materials Science, Mechanics, Metallurgy & Metallurgical Engineering, Meteorology & Atmospheric Sciences, Mineralogy, Nuclear Science & Technology,  Optics, Physics, Robotics, Telecommunications, Thermodynamics | Physics |
| Agriculture, Ecology, Environmental Sciences & Ecology,  Geology, Forestry, Paleontology, Physical Geography, Water Resources | Environmental Studies |
| Anthropology, Behavioral Sciences, Biomedical Social Sciences, Business & Economics, Communication, Criminology & Penology, Demography, Development Studies, Economics, Education & Educational Research, Ethic Studies, Family Studies, Government & Law, International Relations, Management, Philosophy, Public Administration, Religion, Social Issues, Social Sciences - Other Topics, Social Work, Sociology, Transportation, Women’s Studies | Social Sciences |
| Archaelology, Area Studies, Art, Arts & Humanities – Other Topics, Classics, History, History & Philosophy of Science, Literature, Linguistics, Urban Studies | Arts and Humanities |
| Allergy, Anesthesiology, Anatomy & Morphology, Antimicrobal Agents & Chemotherapy, Audiology & Speech-Language, Cardiovascular System & Cardiology, Dentistry, Oral Surgery & Medicine, Dermatology, Emergency Medicine, Endocrinology & Metabolism, Gastroenterology & Hepatology, General & Internal Medicine, Genetics & Heredity, Geriatrics & Gerontology, Health Care Sciences & Services, Hematology, Immunology, Infectious Diseases, Medical Ethics, Medical Laboratory Technology, Integrative & Complementary Medicine, Neurosciences & Neurology, Nursing, Nutrition & Dietetics, Obstetrics & Gynecology, Oncology, Ophthalmology, Orthopedics, Otorhinolaryngology, Pathology, Pediatrics, Pharmacology & Pharmacy, Physiotherapy, Physiology, Psychiatry, Psychology, Public, Environmental & Occupational Health, Radiology, Nuclear Medicine & Medical Imaging, Research & Experimental Medicine, Respiratory System, Rheumatology, Sport Sciences, Substance Abuse, Surgery, Toxicology, Transplantation, Tropical Medicine, Urology & Nephrology, Virology | Health |
| Automation & Control Systems, Computer Science, Information Science & Library Science, Mathematics, Medical Informatics, Operations Research & Management Science, Remote Sensing | Computing and Mathematics |
| Biochemistry & Molecular Biology, Biodiversity & Conservation, Biotechnology & Applied Microbiology, Cell Biology, Developmental Biology, Entomology, Evolutionary Biology, Fisheries, Life Sciences & Biomedicine, Marine & Freshwater Biology, Mathematical & Computational Biology, Microbiology,  Microscopy, Mycology, Oceanography, Paleooceanography, Parasitology, Plant Sciences, Reproductive Biology, Veterinary Sciences, Zoology | Biology |
| Chemistry, Crystallography, Electrochemistry, Food Science & Technology, Polymer Science, Spectroscopy | Chemistry |
| Science and Technology – other topics | Science and Technology |
