# Supplementary material for: Declared funding and authorship by alcohol industry actors in the scientific literature: a bibliometric study
Source: Eur J Public Health. 2020 Sep 17;30(6):1193–200. doi: 10.1093/eurpub/ckaa172 (PMC7733050; doi:10.1093/eurpub/ckaa172)
Supplement: ckaa172_supplementary_data [file ckaa172_supplementary_data.zip › ejph-2020-02-om-0159-File006.docx]

**Supplementary Table S2: Type of Support Declared by all records and overlapping records**

| **Type of Funder and Number of Records** | **Funding** | **Materials** | **Human Resources** |
| --- | --- | --- | --- |
| Company (n=5138) | 4870 (95%) | 221 (4%) | 52 (1%) |
| Organisation (n=1493) | 1343 (90%) | 100 (7%) | 67 (4%) |
| Either or both (n=6613) | 6201 (94%) | 315 (5%) | 117 (2%) |

NB. Some references were identified by more than one search
