# Supplementary material for: Declared funding and authorship by alcohol industry actors in the scientific literature: a bibliometric study
Source: Eur J Public Health. 2020 Sep 17;30(6):1193–200. doi: 10.1093/eurpub/ckaa172 (PMC7733050; doi:10.1093/eurpub/ckaa172)
Supplement: ckaa172_supplementary_data [file ckaa172_supplementary_data.zip › ejph-2020-02-om-0159-File007.docx]

**Supplementary Table S3: Subject areas covered by duplicates and non-duplicate records**

| **Subject area** | **Company duplicates (n=177)** | **Organisation duplicates (n=283)** | **All duplicates (n=450)** | **Company non-duplicates (n=10837)** | **Organisation non-duplicates**  **(n=2205)** | **All non-duplicates (n=13031)** |
| --- | --- | --- | --- | --- | --- | --- |
| Biology | 84 (47%) | 84 (30%) | 167 (37%) | 4752 (44%) | 499 (23%) | 5248 (40%) |
| Chemistry | 74 (42%) | 199 (70%) | 263 (58%) | 2854 (26%) | 824 (37%) | 3674 (28%) |
| Environmental Studies | 22 (12%) | 125 (44%) | 143 (32%) | 1057 (10%) | 487 (22%) | 1544 (12%) |
| Health | 30 (17%) | 44 (16%) | 71 (16%) | 2600 (24%) | 1040 (47%) | 3636 (28%) |
| Science and Technology | 25 (14%) | 8 (3%) | 33 (7%) | 657 (6%) | 53 (2%) | 710 (5%) |
| Physics | 14 (8%) | 4 (1%) | 18 (4%) | 1796 (17%) | 62 (3%) | 1856 (14%) |
| Computing and Mathematics | 1 (0.5%) | 2 (0.7%) | 3 (0.7%) | 149 (1%) | 8 (0.4%) | 156 (1%) |
| Social Sciences | 2 (1%) | 0 | 2 (0.4%) | 255 (2%) | 75 (3%) | 330 (3%) |
| Arts and Humanities | 0 | 0 | 0 | 62 (0.6%) | 1 (0.05%) | 63 (0.5%) |

NB. Some references were identified by more than one search
