# Supplementary material for: Declared funding and authorship by alcohol industry actors in the scientific literature: a bibliometric study
Source: Eur J Public Health. 2020 Sep 17;30(6):1193–200. doi: 10.1093/eurpub/ckaa172 (PMC7733050; doi:10.1093/eurpub/ckaa172)
Supplement: ckaa172_supplementary_data [file ckaa172_supplementary_data.zip › ejph-2020-02-om-0159-File008.docx]

**Supplementary Table S4: References tested for retrieval in Web of Science**

| **Article Reference** | **Identified by the searches** | **Databases available from** | **Text in the full journal article indicating company or organisation support** | **Text in the bibliographic record** | **Explanation for lack of retrieval** |
| --- | --- | --- | --- | --- | --- |
| Appleton A, James R, Larsen J. (T)he Association between Mental Wellbeing, Levels of Harmful Drinking, and Drinking Motivations: A Cross-Sectional Study of the UK Adult Population. Int J Env Res Pub He. 2018;15(7). | Yes | SCI, SSCI | **Funding**: This research was funded by ***Drinkaware***. **Acknowledgments:** We thank YouGov for their work in collecting the data used in the study.  **Conflicts of Interest**: ***Drinkaware*** is primarily funded by voluntary and unrestricted donations from UK alcohol producers, retailers and supermarkets, based on an agreement between the UK government and the alcohol industry. All authors (A.A., R.J., and J.L.) undertook the analysis and write-up as employees at *Drinkaware* and members of the Research and Impact team. | **Organisation: *Drinkaware***  **Funding Agency**: ***Drinkaware***.  **Funding text**: This research was funded by ***Drinkaware****.* | NA |
| Bewick BM, West RM, Barkham M, Mulhern B, Marlow R, Traviss G, et al. The Effectiveness of a Web-Based Personalized Feedback and Social Norms Alcohol Intervention on United Kingdom University Students: Randomized Controlled Trial. Journal of Medical Internet Research. 2013;15(7):106-16. | No | SCI, SSCI | **Acknowledgments**: We thank participants, student union executive members, and university staff who gave their time to be involved in this project. We also thank Jane Cahill as the independent researcher responsible for stratification design and implementation and randomization of the participants. The project was funded by a research grant from the ***European Research Advisory Board*** (Grant 2005-EA0508).  **Conflicts of Interest:** In the past, Bewick, as keynote speaker, has received reimbursement of travel expenses from *Anheuser-Busch* and Noctis. | **Funding Agency**: ***European Research Advisory Board*** **Funding Text**: We thank participants, student union executive members, and university staff who gave their time to be involved in this project. We also thank Jane Cahill as the independent researcher responsible for stratification design and implementation and randomization of the participants. The project was funded by a research grant from the ***European Research Advisory Board*** (Grant 2005-EA0508). | NA |
| Foxcroft DR, Moreira MT, Almeida Santimano NML, Smith LA. Social norms information for alcohol misuse in university and college students (Review). Cochrane Database of Systematic Reviews. 2015 | Yes | SCI, SSCI | **Sources of support:** Oxford Brookes University, UK, FCT—Fundação Ciência e Tecnologia, Portugal, AERC—Alcohol Education and Research Council, UK, ***ERAB—European Research Advisory Board***, Belgium.  **Declarations of interest:** Oxford Brookes University has received funding from the alcohol industry for prevention programme development and training. No conflict of interest has been perceived between the funding provided and this Cochrane review. | **Funding Agency**: Oxford Brookes University, UK, FCT- Fundação Ciência e Tecnologia, Portugal, AERC- Alcohol Education and Research Council, UK, ***ERAB- European Research Advisory Board***, Belgium.  **Funding Text**: Internal sources: Oxford Brookes University, UK, External sources: FCT – Fundacao Ciencia e Tecnologia, Portugal, AERC – Alcohol Education and Research Council, UK and ***ERAB – European Research Advisory Board***, Belgium. | NA |
| M T. Moreira, R Oskrochi, D R. Foxcroft. Personalised Normative Feedback for Preventing Alcohol Misuse in University Students: Solomon Three-Group Randomised Controlled Trial. Plos One. 2012;7:10. | Yes | SCI, SSCI | **Funding:** MTM was supported by a fellowship from the Portuguese Foundation for Science and Technology. Other parts of this study were supported by Alcohol Research UK and the ***European Foundation for Alcohol Research***. The funders had no role in study design, data collection and analysis, decision to publish, or preparation of the manuscript. No additional external funding was received for this study.  **Competing Interests**: MTM and RO declare no competing interests. DRF declares that his Department has received funding from the alcohol industry for prevention projects, and that he is a Trustee of the alcohol-industry funded ***Drinkaware*** Trust. This does not alter the authors’ adherence to all the PLoS ONE policies on sharing data and materials. | **Funding Agency**: Portuguese Foundation for Science and Technology, Alcohol Research UK, ***European Foundation for Alcohol Research***.  **Funding Text**: MTM was supported by a fellowship from the Portuguese Foundation for Science and Technology. Other parts of this study were supported by Alcohol Research UK and the ***European Foundation for Alcohol Research***. The funders had no role in study design, data collection and analysis, decision to publish, or preparation of the manuscript. No additional external funding was received for this study. | NA |
| Wallace P, Murray E, McCambridge J, Khadjesari Z, White IR, Thompson SG, et al. On-line randomized controlled trial of an internet based psychologically enhanced intervention for people with hazardous alcohol consumption. PLoS One. 2011;6(3):e14740. | Yes | SCI, SSCI | **Funding:** This study was funded by the National Prevention Research Initiative, which includes the following funding partners: British Heart Foundation; Cancer Research UK; Department of Health; Diabetes UK; Economic and Social Research Council; Medical Research Council; Research and Development Office for the Northern Ireland Health and Social Services; Chief Scientist Office, Scottish Executive Health Department; and the Welsh Assembly Government. IRW and SGT are funded by the UK Medical Research Council (grant codes U.1052.00.006 and U.1052.00.001). The Alcohol Education and Research Council provided additional funding to assist with developing the intervention site. The funders had no role in study design, data collection and analysis, decision to publish, or preparation of the manuscript. Competing Interests: Since June 2009, PGW has received payments from the charity ***Drinkaware*** in his capacity as its Chief Medical Advisor. PGW has no other competing interests and the authors confirm that this does not alter their adherence to all the PLoS ONE policies on sharing data and materials. | **Funding Agency:** Diabetes UK, British Heart Foundation, Cancer Research UK, Diabetes UK, Economic & Social Research Council (ESRC), Medical Research Council UK (MRC), Research and Development Office for the Northern Ireland Health and Social Services, Chief Scientist Office, Scottish Executive Health Department Welsh Assembly Government,  Alcohol Education and Research Council, ***Drinkaware****.*  **Funding Text**: This study was funded by the National Prevention Research Initiative, which includes the following funding partners: British Heart Foundation; Cancer Research UK; Department of Health; Diabetes UK; Economic and Social Research Council; Medical Research Council; Research and Development Office for the Northern Ireland Health and Social Services; Chief Scientist Office, Scottish Executive Health Department; and the Welsh Assembly Government. IRW and SGT are funded by the UK Medical Research Council (grant codes U.1052.00.006 and U.1052.00.001). The Alcohol Education and Research Council provided additional funding to assist with developing the intervention site. The funders had no role in study design, data collection and analysis, decision to publish, or preparation of the manuscript.  Since June 2009, PGW has received payments from the charity ***Drinkaware*** in his capacity as its Chief Medical Advisor. PGW has no other competing interests and the authors confirm that this does not alter their adherence to all the PLoS ONE policies on sharing data and materials. | NA |
| Sulkunen P. Knowledge is power, and power needs knowledge. Int J Alcohol Drug R. 2016;5(1):11-+. | No | Emerging Sources Citation Index 2015- | No relevant text | No relevant text | No relevant text to search. |
| Anderson P, Rehm J. Evaluating Alcohol Industry Action to Reduce the Harmful Use of Alcohol. Alcohol and Alcoholism, 2016, 51(4)383-7. | No | SCI, SSCI | **FUNDING**: This editorial did not receive any financial support  **CONFLICT OF INTEREST STATEMENT:** P.A. received reimbursement of the costs of his attendance to give a presentation on trends in research on health and alcohol to the Global Advisory Council of ***AB InBev*** in London, July 2015 (presentation available on request). No potential conflict of interest stated for J.R. | No relevant text because as the "Funding" statement states: no funding or sponsoring of the study, information available in the "Conflict of Interest" is ignored. | No relevant text to search. |
| Poikolainen K. Does the Tail Wag the Dog? Abstainers, Alcohol Dependence, Heavy Episodic Drinkers and Total Alcohol Consumption. Alcohol and Alcoholism, 2017, 52(1)80-83. | No | SCI, SSCI | **CONFLICT OF INTEREST STATEMENT**: Fee received for proof reading a report. Panimo- ja virvoitusjuoma- teollisuusliittory (The Federation of the Brewing and Soft Drinks Industry, Finland). | No relevant text because the conflict of Interest/Declaration does not contain direct FUNDED BY OR SUPPORTED BY information. | No relevant text to search. |
| Mukamal KJ, Clowry CM, Murray MM, Hendriks HFJ, Rimm EB, Sink KM, et al. Moderate Alcohol Consumption and Chronic Disease: The Case for a Long-Term Trial. Alcoholism-Clinical and Experimental Research. 2016;40(11):2283-91. | No | SCI | **ACKNOWLEDGMENTS**: This work was supported by grants U13AA023452, U34AA023258, and U10AA025286 from the National Institute on Alcohol Abuse and Alcoholism.  **DISCLOSURES**: Drs. Hendriks and Dragsted have conducted short-term feeding studies with alcohol at institutions (TNO, University of Copenhagen) that have received partial support from members of the ***alcohol industry***. Dr. Krystal has served on the advisory boards and/or holds stock in several companies with interests in psychiatric disorders. | No relevant text because no specific companies named in the article and the term ‘alcohol industry’ is not in any database field.  **Funding Agency:** United States Department of Health & Human Services  National Institutes of Health (NIH) - USA  NIH Fogarty International Center (FIC), United States Department of Health & Human Services  National Institutes of Health (NIH) - USA  NIH National Center for Advancing Translational Sciences (NCATS), United States Department of Health & Human Services  National Institutes of Health (NIH) - USA  NIH National Institute on Alcohol Abuse & Alcoholism (NIAAA) | No relevant text to search. |
| Thom B. Good practice in school based alcohol education programmes. Patient Education and Counseling. 2017;100:S17-S23. | No | SCI, SSCI | **Role of funding and conflict of interest** This paper was funded by ***IARD*** as part of a project to develop an Alcohol Education Guide website. The author was part of a team considering criteria for good practice in developing and implementing school-based alcohol education programmes. An initial draft paper reviewed the literature in more detail. This paper is based on that. IARD did not contribute to the first draft paper or to this paper in anyway. The paper is solely the work of the author. The author is a member of ***IARD*** research advisory group. | **Funding Agency: IARD.**  **Funding Text**: This paper was funded by **IARD** as part of a project to develop an Alcohol Education Guide website. The author was part of a team considering criteria for good practice in developing and implementing school-based alcohol education programmes. An initial draft paper reviewed the literature in more detail. This paper is based on that. ***IARD*** did not contribute to the first draft paper or to this paper in any way. The paper is solely the work of the author. | ***International Alliance for Responsible Drinking*** only searched in full not as acronym as ***IARD*** identified too many irrelevant records |
